# Osler‐Weber‐Rendu syndrome: A case report on a rare vascular malformation presented with lower gastrointestinal bleeding

**DOI:** 10.1002/ccr3.6965

**Published:** 2023-02-15

**Authors:** Sulav Pyakurel, Sujan Bohara, Sanjeet Bhattarai, Sachit Regmi, Samikshya Karki, Mahima Adhikari, Bibek Gurung, Umid Shrestha

**Affiliations:** ^1^ Department of Nephrology Nepal Mediciti Hospital Lalitpur Nepal; ^2^ Department of General and Gastrointestinal Surgery Nepal Mediciti Hospital Lalitpur Nepal; ^3^ Department of Pulmonary Medicine Nepal Mediciti Hospital Lalitpur Nepal; ^4^ Department of Gastrointestinal Medicine Nepal Mediciti Hospital Lalitpur Nepal; ^5^ Spinal Injury Rehabilitation Center Kavre Nepal; ^6^ Department of Radiology Nepal Mediciti Hospital Lalitpur Nepal

**Keywords:** arteriovenous malformation, hereditary hemorrhagic telangiectasia, Osler‐Weber‐Rendu syndrome, vascular dysplasia

## Abstract

Osler‐Weber‐Rendu syndrome is an uncommon vascular disorder inherited as an autosomal dominant trait with varying penetrance and expression. A multidisciplinary approach is used for a detailed diagnostic workup and management based on the patient's symptoms at presentation.

## Graphical abstract

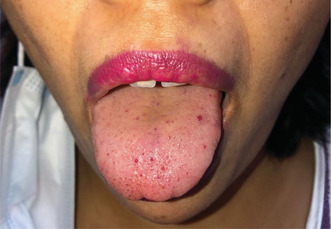

## 1 INTRODUCTION

Osler‐Weber‐Rendu syndrome, also known as hereditary hemorrhagic telangiectasia (HHT), is a rare autosomal dominant genetic disorder with a prevalence of 1:5000 to 1:8000. HHT gene pathogenic variants lead to the development of aberrant vascular structures that range from dilated micro‐vessels to large arteriovenous malformations (AVMs).^1^ Abnormal angiogenesis occurs in the skin, mucous membranes, and often in visceral organs such as the lungs, liver, and brain.^2^ Thus, the syndrome is characterized by epistaxis (as an initial manifestation) along with a cutaneo‐mucous manifestation and often in association with the clinical spectrum of various malformations of the liver, lungs, and gastrointestinal tract, which may be asymptomatic or exhibit a wide range of clinical symptoms.^3^, ^4^

Visceral AVMs of the liver, lungs, and central nervous system are common and usually asymptomatic; complications include high‐output heart failure, portal hypertension, liver failure, hemoptysis, polycythemia, cerebral abscess, and stroke. The Curaçao diagnostic criteria are used for the diagnosis of HHT.^5^ The management of HHT should include a comprehensive diagnostic workup and screening for iron deficiency, hepatic AVMs, pulmonary AVMs, and cerebral AVMs, among other family members. Generally, the patient's clinical spectrum and specific vascular abnormalities are the primary factors considered in treatment.^1^, ^5^, ^6^

We here describe a female who presented with rectal bleeding associated with constipation and significant past and family history and was diagnosed with a rare genetic syndrome, Osler‐Weber‐Rendu syndrome, via clinical history, examination findings, and radiological investigation.

## 2 CASE PRESENTATION

A 51‐year‐old female, non‐diabetic, normotensive, with no associated chronic medical illnesses, presented to our gastroenterology outpatient care with a history of fresh rectal bleeding that was associated with a history of constipation. She also detailed a history of recurrent nose bleeds since childhood, which occurred frequently during the summer season and were previously treated with ablative therapies in local medical centers. Multiple clinical visits for iron deficiency anemia (IDA), which was previously treated with oral iron supplements, were documented in her medical records. She denied having experienced any of the following symptoms: shortness of breath, chest pain, joint pain or swelling, easy bruising, delayed bleeding stoppage, blood transfusion, weight loss, night sweats or fever. Her family history noted similar symptoms in her mother, along with telangiectatic lesions on the lips; her brother likewise has lesions on the lips, as do her son and daughter. She was a non‐smoker and did not consume alcohol. She was a P3L2A1 female and had previously undergone a total abdominal hysterectomy due to an ectopic pregnancy many years ago.

**FIGURE 1:**
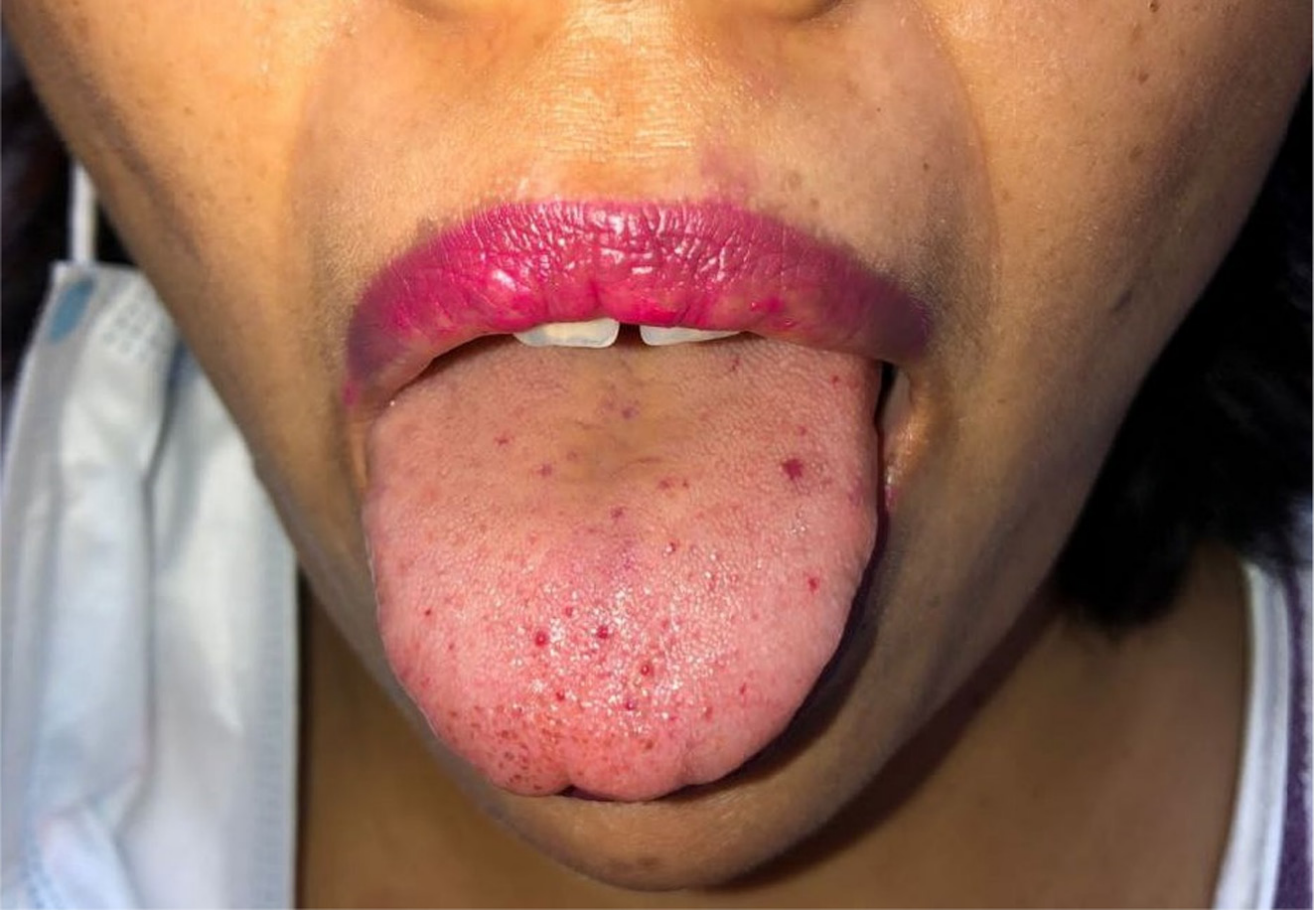
Photograph showing multiple foci of telangiectasia over the tongue of patients.

Clinical examination revealed conjunctival pallor along with multiple telangiectatic lesions on the upper and lower lips, in the mucosa of the lower lip, as well as on the tongue (Figure 1). An abdominal examination revealed no sign of hepatosplenomegaly or abdominal bruit. Likewise, a chest auscultation elicited no aberrant auscultatory findings.

**FIGURE 2:**
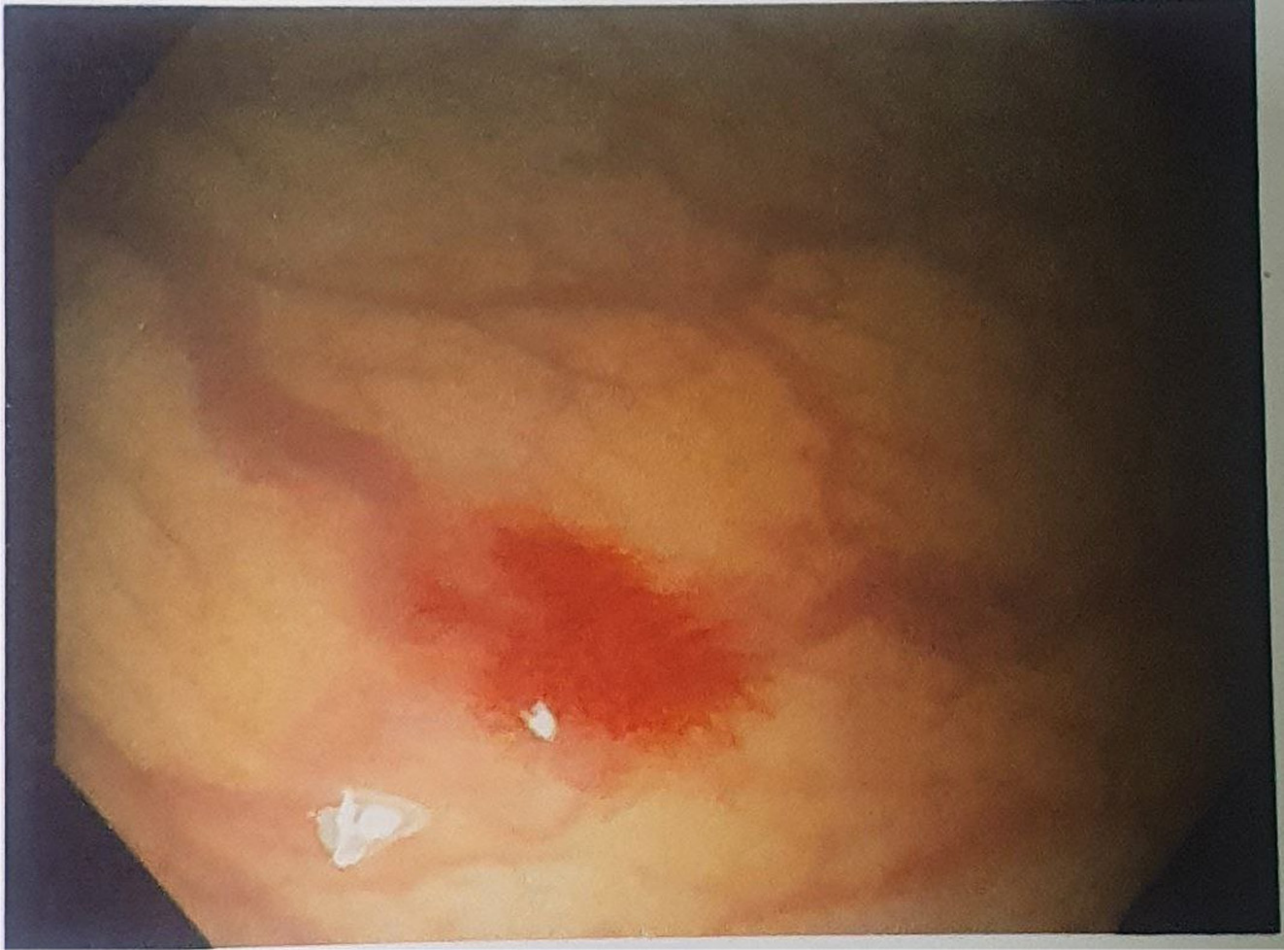
Colonoscopic image showing area of angiodysplasia in the rectal mucosa.

**FIGURE 3:**
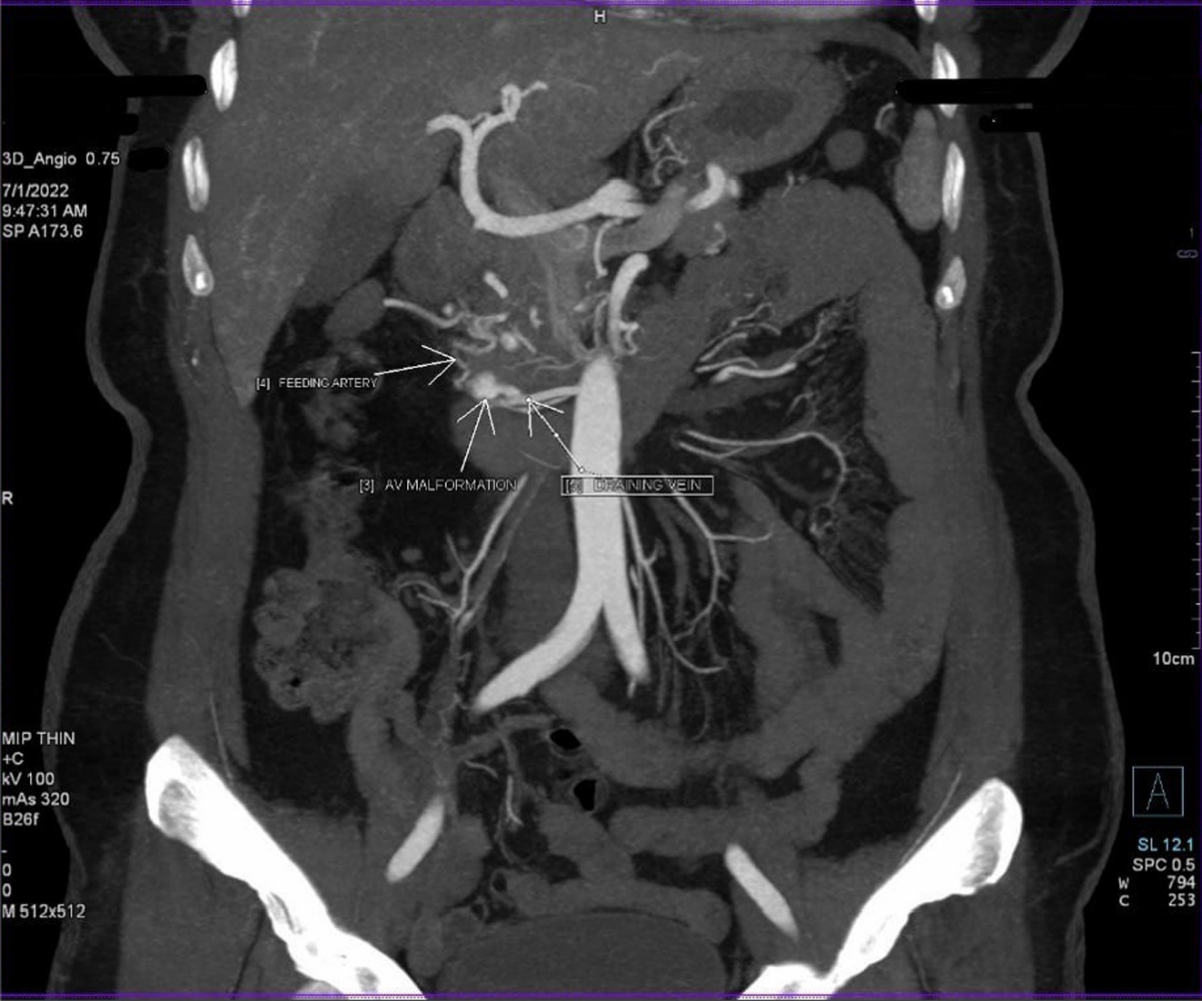
Computed tomography angiogram showing AVM with feeding artery as superior mesenteric artery, nidus around uncinate process, and draining to superior mesenteric vein.

Following that, she was sent for her routine blood work‐up, which came out to be normal except for the hemoglobin (11.1 g%), RBC count (4.30 m/mm3), and PCV (34.2%). Furthermore, she was advised to have a colonoscopy (Figure 2) in light of fresh rectal bleeding and anemia, which showed a small area of angiodysplasia in the rectum without active bleeding and a small internal hemorrhoid. A computed tomography angiogram of the abdominal aorta and its branches (Figure 3) revealed liver hemangiomas and multiple lesions suggestive of AVMs in the liver and pancreas. After all, with all the clinical history, examination, and diagnostic workup, she was eventually diagnosed as having Osler‐Weber‐Rendu Syndrome (Hereditary Hemorrhagic Telangiectasia) after meeting more than three criteria from the international consensus diagnostic criteria^6^ for the syndrome.

Following this, she was counseled about the genetic associations of the disease and the essential steps to take afterward. Iron supplements and advice for follow‐up visits, whether regular or emergency, were given to her before discharge.

One month later, she landed in our emergency room with rectal bleeding once more but normal hemodynamic status, for which she underwent a second colonoscopy. She was admitted to the gastroenterology department this time, and because she had rectal angiodysplasia, she had argon plasma coagulation to control the active bleeding. During her 2‐day hospital stay, she was evaluated by a pulmonary and cardiovascular medicine team for any AVM‐related complications. She completed a 2‐day evaluation before being discharged, and she is currently attending outpatient follow‐up. Her hemoglobin and hematocrit levels were 12.1 g% and 37%, respectively, at her most recent checkup.

## 3 DISCUSSION

Vascular malformations are sporadic and rarely familial in nature, resulting from abnormalities in vascular morphogenesis and/or angiogenesis during early embryonic life (mostly between 4 and 10 weeks of gestation).^2^, ^7^ Among the various arteriovenous malformations (AVM), the most prevalent inherited AVM is Osler‐Weber‐Rendu Syndrome (also known as hereditary hemorrhagic telangiectasia).^7^ HHT is a rare autosomal dominant vascular genetic condition with clinical variation.^2^, ^3^ Three major disease‐associated genes causing HHT are *ENG* (*Endoglin; OMIM NO. 131195*), *ACVRL1* (*Activin A Receptor‐Type II Like 1; OMIM NO. 601284*), and *SMAD4* (*Mothers Against Decapentaplegic Homolog 4; OMIM NO. 600993*), all of which are involved in the transforming growth factor beta (TGF‐β) signaling pathway that is required for the development and maintenance of arteriovenous identity.^8^ HHT comes in two forms: HHT1 and HHT2; HHT1 is more usually associated with the *ENG* gene pathogenic variant along with presentation as lung AVMs, whereas HHT2 is more commonly associated with the *ACVRL1* gene pathogenic variant with presentation as hepatic AVMs as well as late onset.^3^, ^7^ Likewise, patients with pathogenic variants in *MADH4* (which encodes *SMAD4*) present with HHT with Juvenile polyposis.^3^

As a result of the disruption of the TGFβ pathway, aberrant angiogenesis (a modification in the elastic and muscle layers of the vessel walls) occurs, leading to severe fragility and arteriovenous malformations, rendering them more susceptible to spontaneous ruptures and injuries.^8^ The majority present with mucocutaneous telangiectasias in association with pulmonary (30%–50% of patients), hepatic (40% of patients), GI (15%–30%) and CNS (5%–20%) telangiectasias. Cardiac or hepatic failure may occur occasionally secondary to a hepatic malformation, though they are found to have a low propensity to bleed.^2^ While epistaxis, mucocutaneous telangiectasia, and iron deficiency anemia secondary to blood loss are common features, visceral AVMs are usually silent but can result in major pathology.^6^ Our patient initially presented with nasal bleeding in her early childhood, which is usually managed with ablative therapies. Likewise, during her first and second presentations, lower gastrointestinal bleeding remained the primary complaint.

The diagnosis of HHT is based on clinical suspicion and a supportive family history of the disease. The second International Guideline on HHT recommends making (or excluding) the diagnosis using the Curaçao criteria (Table 1) and/or identifying pathogenic variants in one of the HHT genes.^6^ Our patient fulfilled all of the criteria for an HHT diagnosis.

**TABLE 1:** International consensus diagnostic criteria (the Curaçao Diagnostic Criteria).

Components	
1	Spontaneous and recurrent epistaxis
2	Multiple mucocutaneous telangiectasia at characteristic sites
3	Visceral involvement (e.g., gastrointestinal telangiectasia; pulmonary, cerebral, or hepatic AVMs)
4	A first‐degree relative with HHT
Interpretation	
3 or 3+ criteria present→ Definite HHT 2 criteria present→ Suspected HHT 1 criteria present → Unlikely HHT	

Therefore, screening for AVMs, particularly pulmonary and cerebral AVMs, should be considered in adults; there is limited evidence of benefit for screening in children compared to the risk of radiation exposure from certain imaging studies. However, multiple case studies and reports have shown that spinal AVMs and CVMs in children can have fatal hemorrhagic consequences. Rarely, spontaneous CVM resolution has been documented.^6^

Because visceral telangiectasia and AVMs are predisposed to severe complications, an early diagnostic work‐up (Table 2) with high sensitivity is essential.^1^, ^6^, ^9^, ^10^ Our patient had a colonoscopy, which revealed angiodysplasia, as well as a CT‐angiogram, which revealed an arteriovenous malformation between the superior mesenteric artery and superior mesenteric vein.

**TABLE 2:** Diagnostic work‐up of different spectrum of HHT.

Components	Work‐up
Nasal telangiectasia	Complete blood count and peripheral blood smear; serum ferritin and iron level (Note: Serum iron levels are generally worked up if ferritin is not reduced and the patient is anemic.)
GI AVMs	Esophagogastroduodenoscopy(EGD; first‐line diagnostic approach) and colonoscopy; fresh occult blood test
Pulmonary AVMs	Transthoracic contrast echocardiography; if positive, confirm with high‐resolution thoracic CT
Hepatic AVMs	Doppler USG for screening; triple helical CT if there is clinical suspicion of complications from hepatic AVMs such as high‐output congestive heart failure or portal hypertension; contrast abdominal MRI
CNS/Brain AVMs	Brain MRI with or without gadolinium
Genetic testing	Screening for *ENG* and *ACVRL1*, and/or *SMAD4*; colonoscopy at 15 years for *SAMD4‐HHT*

Although genetic testing is not required to make the diagnosis of HHT, the second International Guideline suggests genetic testing for all individuals with HHT, as it may facilitate family testing and additional evaluations (e.g., screening colonoscopies for individuals with pathogenic variants in the *SMAD4* gene). Genetic testing can also be used to establish the diagnosis in individuals with suspected HHT who do not meet clinical criteria. Additionally, the discovery rate for a causal or possibly causative pathogenic variant in an exon or intron/exon border of *ENG, ACVRL1*, or *SMAD4* is 97% for patients who meet the Curaçao diagnostic criteria for HHT.^6^ The authors, however, did not use genetic testing to diagnose this condition.

Treatment focuses solely on symptom‐based treatment utilizing interdisciplinary approaches (including cardiologists, pulmonologists, gastroenterologists, and hepatologists as well as specialists in interventional radiology, genetics, and hematology). This lessens the significant morbidity and mortality brought on by potential HHT complications in the future.^5^ Thus, management for HHT is solely based on symptoms at presentation, as in Table 3.^1^, ^6^, ^9^, ^10^, ^11^ During the second presentation of our patient, following angiodysplasia in the colon during colonoscopy, she was managed with argon plasma coagulation as well as evaluated by the multidisciplinary team for any new manifestation of the syndrome.

**TABLE 3:** HHT components, their manifestations, and the management strategies.

Components	Manifestations	Management
GI AVM	GI bleeding	Iron supplementation (oral or parenteral) and/or blood transfusion Oral estrogen or progesterone therapy and tranexamic acid; Bevacizumab (anti‐VEGF monoclonal antibody); Argon plasma coagulation for colonic angiodysplasia
Hepatic AVM	Arteriovenous / arterioportal / portovenous shunts → leading to high output cardiac failure, liver cirrhosis and portal hypertension leading to variceal bleed.	Symptomatic high output cardiac failure due to AVMs→ IV Bevacizumab (anti‐VEGF monoclonal antibody; for patient unresponsive to first‐line management) Orthotopic liver transplantation represents the only definitive curative option for hepatic AVMs in HHT; Hepatic embolization → contraindicated due to life‐threatening hepatic infarction.
Nasal telangiectasia	Epistaxis	Air humidification, nasal packing Medical modalities: Angiogenic inhibitors (Bevacicumab), Hormonal therapies (combined estrogen and progesterone contraception), antifibrinolytics (tranexamic acid) Surgical modalities: Laser coagulation, septal dermoplasty, nasal arterial embolization, submucosal radiofrequency
Pulmonary AVM	Neurological manifestations secondary to PAVM	Transcatheter embolization for PAVMs with feeding artery diameters greater than 2–3 mm Amplatzer vascular plugs for larger, complex and multiple AVMs For neurological sequelae/manifestation such as cerebral abscess → prophylactic antibiotics
CNS AVM	Stroke; intracerebral hemorrhage, epilepsy, transient ischemic attack or spinal hemorrhage.	Depending on size, location, and symptoms, pen microsurgical excision or surgery, embolotherapy or stereotactic radiosurgery (for inoperable lesions) is often indicated.

## 4 CONCLUSION

HHT is a rare multi‐systemic genetic disorder with aberrant angiogenesis. For the specific management, which is primarily symptom‐based, a multidisciplinary approach is used, as well as providing follow‐up to monitor the development of new complications. Clinicians should be aware of this rare disease and consider it as part of the differential diagnosis whenever a patient clinically presents with lower gastrointestinal bleeding with a significant family history.

## AUTHOR CONTRIBUTIONS

**Sulav Pyakurel:** Conceptualization; methodology; writing – original draft. **Sujan Bohara:** Conceptualization; methodology; resources; writing – review and editing. **Sanjeet Bhattarai:** Conceptualization; methodology; writing – original draft. **Sachit Regmi:** Conceptualization; methodology; writing – original draft. **Samikshya Karki:** Resources; writing – review and editing. **Mahima Adhikari:** Data curation; investigation. **Bibek Gurung:** Data curation; investigation. **Umid Shrestha:** Supervision.

## FUNDING INFORMATION

All authors declare that they have not received any grants or funding for this manuscript.

## CONFLICT OF INTEREST STATEMENT

All the authors declare that they have no conflicts of interest.

## ETHICS STATEMENT

Not required for the publication of this manuscript.

## CONSENT

Written informed consent was obtained from the patient for publication of this case report and accompanying images. A copy of the written consent is available for review by the editor‐in‐chief of this journal on request.

## Data Availability

The data used to support the findings of this study are available from the corresponding author upon request.

## References

[1] Govani FS , Shovlin CL . Hereditary haemorrhagic telangiectasia: a clinical and scientific review. Eur J Hum Genet. 2009;17(7):860‐871. doi:10.1038/ejhg.2009.35 19337313 PMC2986493

[2] Boon LM , Ballieux F , Vikkula M . Pathogenesis of vascular anomalies. Clin Plast Surg. 2011;38(1):7‐19. doi:10.1016/j.cps.2010.08.012 21095468 PMC3031181

[3] Brouillard P , Vikkula M . Genetic causes of vascular malformations. Hum Mol Genet. 2007;16(R2):140‐149. doi:10.1093/hmg/ddm211 17670762

[4] Silva BM , Hosman AE , Devlin HL , Shovlin CL . Lifestyle and dietary influences on nosebleed severity in hereditary hemorrhagic telangiectasia. Laryngoscope. 2013;123(5):1092‐1099. doi:10.1002/lary.23893 23404156

[5] Kritharis A , Al‐Samkari H , Kuter DJ . Hereditary hemorrhagic telangiectasia: diagnosis and management from the hematologist's perspectives. Haematologica. 2018;103:1433‐1443. doi:10.3324/haematol.2018.193003 29794143 PMC6119150

[6] Faughnan ME , Mager JJ , Hetts SW , et al. Second international guidelines for the diagnosis and management of hereditary hemorrhagic telangiectasia. Ann Intern Med. 2020;173(12):989‐1001. doi:10.7326/M20-1443 32894695

[7] Borst AJ , Nakano TA , Blei F , Adams DM , Duis J . A primer on a comprehensive genetic approach to vascular anomalies. Front Pediatr. 2020;8:579591. doi:10.3389/fped.2020.579591 33194911 PMC7604490

[8] Fernández‐L A , Sanz‐Rodriguez F , Blanco FJ , Bernabéu C , Botella LM . Hereditary hemorrhagic telangiectasia, a vascular dysplasia affecting the TGF‐β signaling pathway. Clin Med Res. 2006;4(1):66‐78. doi:10.3121/cmr.4.1.66 16595794 PMC1435660

[9] Garg N , Khunger M , Gupta A , Kumar N . Optimal management of hereditary hemorrhagic telangiectasia. J Blood Med. 2014;5:191‐206. doi:10.2147/JBM.S45295 25342923 PMC4206399

[10] McDonald J , Stevenson DA . Hereditary hemorrhagic telangiectasia. In: Adam MP , Everman DB , Mirzaa GM , et al., eds. GeneReviews® [Internet]. University of Washington, Seattle; 2000. 20301525

[11] Tortora A , Riccioni ME , Gaetani E , Ojetti V , Holleran G , Gasbarrini A . Rendu‐Osler‐Weber disease: a gastroenterologist's perspective. Orphanet J Rare Dis. 2019;4:14‐17. 10.1186/s13023-019-1107-4 PMC6555961 31174568

